# Exploring the connection between authoritarian-benevolent leadership and craftsman spirit: the mediating role of loyalty

**DOI:** 10.3389/fpsyg.2026.1456088

**Published:** 2026-03-23

**Authors:** Huanhuan Zhang, Fan Li

**Affiliations:** 1 School of Tourism, Xinyang Agriculture and Forestry University, Xinyang, China; 2 School of Education, Huazhong University of Science and Technology, Wuhan, China; 3 School of Psychology, Henan University, Kaifeng, China

**Keywords:** benevolent leadership, authoritarian leadership, loyalty, craftsman spirit, authoritarian-benevolent leadership

## Abstract

Guided by social exchange theory, this study examines how the dual leadership behavior of authoritarian-benevolent leadership in mentorship affects craftsman spirit within China’s manufacturing sector amidst its transformation and upgrading. A theoretical model positioning loyalty as a mediator was developed to elucidate how different configurations of benevolent and authoritarian leadership influence craftsman spirit through reciprocal social exchanges. Using polynomial regression and response surface analysis on survey data from 343 master-apprentice dyads, the findings indicate that: (1) when benevolence and authority are aligned, high levels of both yield no significant advantage over low levels of both in promoting craftsman spirit; (2) when benevolence and authority are misaligned, high benevolence coupled with low authority more effectively fosters craftsman spirit than the reverse; (3) loyalty mediates the relationship between high benevolence-low authority leadership and craftsman spirit. These results suggest that manufacturing firms should incorporate ambidextrous leadership into master selection and training, while establishing institutionalized master-apprentice pairing mechanisms and emotional bank account management to systematically cultivate craftsman spirit.

## 1 Introduction

The Craftsman spirit refers to the professional attitudes characterized by conscientiousness, dedication, and a behavioral inclination toward persistent care, excellence, and positive innovation exhibited by highly skilled employees throughout their employment. Its core essence lies in the values of dedication, excellence, and innovation (Ye et al., 2020). Craftsman spirit manifests distinct characteristics across cultural contexts, embodying both national identity and contemporary relevance. Unlike Western craftsman spirit that prioritizes standardization and efficiency, the Chinese concept of craftsman spirit emphasizes more adaptable and human-centric dimensions. Its inherent philosophy of “creating in harmony with natural principles” underscores the importance of following natural laws in design and manufacturing while pursuing excellence. This concept represents not only the core requirement for cultivating master craftspeople, but more fundamentally, serves as the essential driving force behind the transformation of “Made in China” into “Created by China.” When it comes to nurturing the craftsman spirit, scholars have explored various avenues, encompassing macro-level factors like the policy system, social environment, and higher education (Xu, 2023). Additionally, scholars have underscored the micro-level significance of leadership styles and their positive impact (Liu et al., 2022). The authoritarian-benevolent leadership style represents a dualistic leadership behavior deeply rooted in the Chinese cultural context (Rosing et al., 2011), characterized by the complementary integration of both benevolent and authoritarian dimensions. Influenced by pan-familism in Chinese organizations, leaders often establish quasi-filial relationships with subordinates, simultaneously demonstrating authority through strict demands and obedience expectations, while showing benevolence through care and support that makes employees feel valued (Hou and Peng, 2019). This unique combination constitutes what is known as the authoritarian-benevolent leadership approach. Authoritarian-benevolent leadership achieves managerial effectiveness through dynamic balancing of seemingly contradictory authoritative and benevolent behaviors (Chen and Weng, 2023). This leadership approach constitutes more than a simple aggregation of behaviors, as its internal configuration differentially impacts employee outcomes. Specifically, high benevolence-low authority combinations prove more effective in stimulating employees’ promotive voice behavior compared to high authority-low benevolence approaches (Chan and Mak, 2012), while moderate balance between authority and benevolence optimally enhances employee innovation performance (Meng et al., 2022). Regarding underlying mechanisms, research has shifted from examining single mediating or moderating variables to exploring more complex pathways, revealing the crucial roles of leader-member exchange relationships, employee psychological states, and individual characteristics in these processes. Current research has not thoroughly investigated the relationship between authoritarian-benevolent leadership and employee craftsman spirit. Given that the transmission of craftsman spirit primarily occurs through familial or mentorship-based systems, and many enterprises have implemented mentorship programs in practice, this study examines typical authoritarian-benevolent scenarios within mentorship systems. It analyzes how such leadership influences craftsman spirit through internal psychological mechanisms like employee loyalty, offering significant theoretical and practical implications for understanding how dual leadership behaviors in Chinese cultural contexts can cultivate high-quality skilled workers.

## 2 Theoretical framework and hypotheses

### 2.1 Social exchange theory

According to social exchange theory, the formation of relationships must be predicated on the ability to provide benefits that the other party requires. This framework operates under the norm of reciprocity, wherein both parties engaged in voluntary interaction anticipate receiving roughly equivalent returns (Baldwin, 1978). When an individual receives assistance without providing commensurate reciprocation, they experience a sense of obligation that motivates them to seek opportunities to fulfill this responsibility, thereby alleviating psychological discomfort (Cropanzano et al., 2001). Through this mechanism, parties gradually establish a stable relationship characterized by mutual trust and willingness for long-term maintenance. The persistence and reinforcement of social exchange relationships depend fundamentally on whether both parties obtain anticipated benefits; failing this condition, such relationships become unsustainable. Social exchange theory thus provides a robust theoretical lens for understanding reciprocal behavior in human societies. In the specific context of the relationship between authoritarian-benevolent leadership and craftsman spirit, the master’s benevolence, operating through the reciprocity principle, evokes the apprentice’s sense of reciprocal obligation and fosters trust, thereby transforming into an intrinsic motivation for pursuing excellence and continuous improvement. Conversely, the authoritative aspect becomes legitimized within this stable exchange framework, perceived as essential investment for skill enhancement rather than mere suppression. Authoritarian-benevolent leadership cultivates craftsman spirit through this integrated process of reciprocal exchange and legitimacy construction. Within this prototypical exchange dynamic, should the apprentice fail to demonstrate loyalty toward the master, this reciprocity-based relationship would prove difficult to sustain. Therefore, this study introduces apprentice loyalty as a mediating mechanism to further investigate the relationship between authoritarian-benevolent leadership and craftsman spirit through the theoretical lens of social exchange theory.

### 2.2 The relationship between authoritarian-benevolent leadership and the craftsman spirit

Against the macroeconomic backdrop of “Made in China 2025” implementation, skills transmission has evolved beyond individual development to carry significant national strategic importance. The mentorship system serves as a crucial foundation for talent development in modern enterprises, playing a pivotal role in skills inheritance, cultural continuity, and innovation advancement. Masters are driven both by traditional responsibilities of knowledge transmission and organizational pressures to cultivate high-quality skilled personnel; apprentices simultaneously seek professional competencies for social mobility and anticipate enhanced career development within the industrial upgrading paradigm. These dual motivations establish the social exchange foundation for master-apprentice relationships (Wu et al., 2019). Within this context, masters provide parental-like guidance for apprentices’ professional and personal lives while expecting loyalty and deference in return (Gelfand et al., 2007), rendering masters’ leadership styles particularly influential on apprentices’ behaviors. The varying degrees to which masters demonstrate benevolent and authoritarian leadership behaviors may consequently exert differential impacts on the development of craftsman spirit.

When masters demonstrate equivalent levels of benevolent and authoritarian leadership behaviors, they may exhibit either high-high or low-low leadership patterns. Under the high-high pattern, masters simultaneously display authoritarian tendencies by controlling work processes through strict supervision, centralized decision-making, and reprimands to ensure complete compliance and high performance (Liu et al., 2021). Apprentices who violate organizational rules face severe admonishment (Jia et al., 2020), which may partially suppress their proactive initiatives. Conversely, these masters also show respect and provide comprehensive support through professional guidance and personal care, thereby fostering apprentices’ sense of reciprocal obligation (Wang and Cheng, 2010) that motivates self-discipline and pursuit of excellence. The flexible adaptation and coordination of these dual styles across different situations enables masters to improve apprentices’ work attitudes and enhance emotional commitment (Grego-Planer, 2022), ultimately eliciting reciprocation through meticulous craftsmanship and active innovation (Lu et al., 2021). Under the low-low pattern, reduced authoritarianism may lead to decreased vigilance and increased negligence, while the absence of benevolent support further reinforces apprentice inertia (Shaw et al., 2020), consequently failing to stimulate innovative initiatives. In such conditions, apprentices perceive insufficient reciprocal exchange, preventing the formation of interdependent social exchange relationships essential for cultivating craftsman spirit. Accordingly, this study proposes a first hypothesis:

*H1*: When masters exhibit consistent benevolent and authoritarian leadership behaviors, the enhancement of apprentices’ craftsman spirit is more pronounced in the high benevolence-high authority leadership style compared to the low benevolence-low authority leadership style.

When masters demonstrate inconsistent levels of benevolent and authoritarian leadership behaviors, they typically exhibit either high benevolence-low authority or high authority-low benevolence patterns. In high benevolence-low authority scenarios, masters foster apprentices’ sense of belonging through care, recognition, and motivation while guiding them toward work objectives via communication and demonstration. This leadership approach facilitates positive social exchange relationships between masters and apprentices (Su et al., 2022), motivating apprentices to reciprocate with positive work behaviors and attitudes to maintain this mutually beneficial dynamic Furthermore, the high benevolence-low authority style minimizes coercive commands while emphasizing relational harmony, whose inclusive nature and low-authority characteristics foster a relaxed, pleasant, and secure atmosphere conducive to apprentices’ innovative initiatives (Lu et al., 2021). Conversely, in high authority-low benevolence situations, masters heavily rely on positional power and regulatory mechanisms to drive task completion through commands, monitoring, and penalties, while providing minimal work support or emotional interaction. This leads to apprentices’ passive compliance driven by authority apprehension rather than genuine acceptance, creating perceptions that masters prioritize personal gains over apprentices’ needs and development (Shaw et al., 2020). Consequently, this leadership pattern inhibits genuine internal identification and work motivation (Jiang et al., 2017), ultimately impeding the development of craftsman spirit. Accordingly, this study proposed a second hypothesis:

*H2*: When masters display inconsistent benevolent and authoritarian leadership behaviors, the enhancement of the craftsman spirit is more significant in the high benevolence-low authority leadership style compared to the low benevolence-high authority leadership style.

### 2.3 The mediating role of loyalty

Loyalty serves as a critical outcome of high-quality exchange relationships, reflecting an individual’s profound emotional attachment to the organization (Masakure, 2016), psychological identification with organizational culture and values (Zhang et al., 2022), and behavioral commitment to remain with the organization long-term (Phuong and Le Ha, 2022). According to the reciprocity principle in social exchange theory, when employees can access and fulfill their valued resources and achievement needs within the organization, they reciprocate with enhanced positive attitudes and organizational loyalty (Khoreva, 2016). Research demonstrates that leadership styles significantly influence the establishment of exchange relationships and consequently affect employee loyalty (Liu et al., 2021). Benevolent and authoritarian leadership behaviors interact synergistically, where their integration through authoritarian-benevolent leadership effectively cultivates subordinate loyalty (Cheng et al., 2002) and promotes employee innovation (Tian and Sanchez, 2017). Under consistent leadership patterns, the high-high configuration demonstrates that although masters’ strong authoritarian behaviors may generate negative impacts on apprentices’ behaviors and attitudes (Schaubroeck et al., 2017), their highly benevolent behaviors effectively mitigate these adverse effects. When masters provide substantial care and support, apprentices develop strong reciprocation beliefs (Farh and Cheng, 2000), and the simultaneous presence of strictness and compassion enhances apprentices’ perception of developmental intent, thereby strengthening loyalty (Liu et al., 2021). Conversely, the low-low configuration indicates minimal master attention and discipline (Shaw et al., 2020), resulting in neither social nor economic exchange between masters and apprentices, thereby preventing the formation of effective exchange relationships.

When masters demonstrate inconsistent levels of benevolent and authoritarian leadership behaviors, under the high benevolence-low authority condition, masters fulfill apprentices’ socioemotional needs by investing substantial socioemotional resources through professional guidance, personal care, and resource support. Apprentices perceive such extra-contractual care as genuine organizational support, thereby developing strong reciprocal intentions and obligation based on reciprocity norms (Chen et al., 2014). This high-quality social exchange relationship, grounded in trust and affection, motivates apprentices to demonstrate loyalty and commitment to their masters (Grego-Planer, 2022). Conversely, under high authority-low benevolence conditions, masters exhibit authoritarian-dominant and even demeaning behaviors. The predominant authoritarian approach, lacking sufficient benevolent buffering, negatively impacts apprentices’ expectations regarding organizational support and career development (Ahmad Bodla et al., 2019), while intensifying their sense of uncertainty and insecurity (Gu et al., 2020; Chiang et al., 2021). Such conditions hinder the formation of stable exchange relationships and ultimately fail to inspire genuine loyalty among apprentices (Asim et al., 2021).

Social exchange theory posits that loyalty plays a crucial role in sustaining and enhancing high-quality exchange relationships (Xie et al., 2012). This loyalty manifests as apprentices’ heightened psychological dependence on their masters and increased behavioral investment in self-improvement. On one hand, apprentices proactively refine their skills to gain master recognition; on the other hand, masters tend to provide loyal apprentices with greater resources and support, thereby motivating reciprocal demonstrations of enhanced work initiative and extra-role efforts, such as increased innovative behavior, fostering a self-reinforcing cycle of reciprocity. Combining the above ideas with hypotheses 1 and 2, this study suggests that loyalty mediates in the relationship between the master’s authoritarian-beneficent leadership behaviors and the apprentice’s craftsman spirit. Accordingly, this study proposes a third hypothesis:

*H3*: Apprentice loyalty mediates the relationship between the master’s authoritarian-benevolent leadership behaviors and the development of the apprentice’s craftsman spirit.

**Figure 1:**
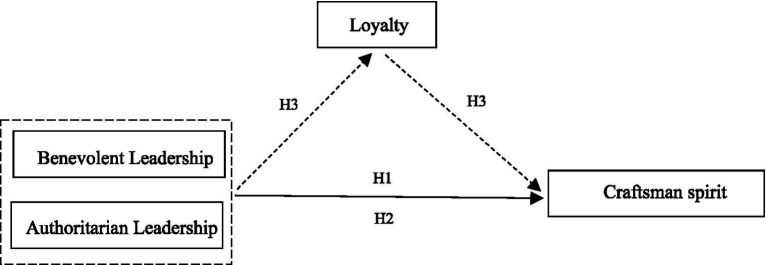
Theoretical model.

Based on the above analyses, this study develops a theoretical model as shown in Figure 1.

## 3 Methods

### 3.1 Participants

In this study, a survey was conducted with members of enterprises located in Zhejiang and Tianjin, encompassing various industries such as machinery processing, energy development, geological exploration, and architectural design. These industries are characterized by their technology-intensive nature, emphasis on standardized operational protocols, and dependence on long-term experience accumulation, with skills transmission heavily reliant on stable mentorship systems. Furthermore, their stringent requirements for process precision, engineering quality, and professional competence align closely with the core principles of craftsman spirit. These distinctive industry characteristics consequently provide an ideal organizational context for examining the relationship between authoritarian-benevolent leadership behavior and craftsman spirit. The survey was conducted in two rounds. During the first round, 450 apprentices were invited to provide their personal basic information and assess their immediate masters’ benevolent and authoritative leadership behaviors. After collecting the questionnaires, 389 valid questionnaires were obtained, resulting in a recovery rate of 86.44%. Questionnaires with random or missing answers were excluded from the analysis. The second round was conducted 2 weeks later. The 389 apprentices who had completed the first round were invited to self-assess their loyalty, while their immediate masters were invited to evaluate their apprentices’ craftsman spirit. A total of 343 valid matched questionnaires were obtained, with a recovery rate of 88.17%.

### 3.2 Instruments

*Craftsman spirit*: the study utilized the revised scale developed by Ye et al. (2018), which comprises three dimensions, namely dedication, excellence and innovation. The scale consists of a total of 25 items, for instance, “The apprentice is passionate about his work and can persevere to accomplish his work goals.” The three dimensions of the scale demonstrated good internal consistency, with Cronbach’s alpha coefficients of 0.75, 0.87 and 0.92, respectively. Respondents rated their agreement with these statements on a on a 6-point Likert scale, with 1 indicating complete non-compliance and 6 indicating full compliance. The Cronbach’s alpha coefficients for love of work, excellence, and innovation in this study were 0.89, 0.82 and 0.95, respectively.

*Benevolent leadership*: the study employed the revised benevolent leadership scale developed by Fu et al. (2013), which comprises five items. Sample questions include, “The master usually asks me about my health.” The scale demonstrated good internal consistency, with a Cronbach’s alpha coefficient exceeding 0.70. Respondents were required to rate their responses on a 6-point Likert scale, with 1 indicating “never” and 6 indicating “always.” The Cronbach’s alpha coefficient for this scale in this study was 0.91.

*Authoritative leadership*: the authoritative leadership scale developed by Cheng et al. (2014) was employed in this study and consists of five items. Sample questions include, “The master is a bit aggressive in front of the apprentice.” The scale demonstrated good internal consistency, yielding a Cronbach’s alpha of 0.88. Respondents rated their responses on a 6-point Likert scale with 1 indicating “never” and 6 indicating “always.” The Cronbach’s alpha coefficient for this scale in this study was 0.82.

*Apprentice loyalty*: the study employed the employee loyalty scale developed by Yao et al. (2008), which comprises two dimensions, namely attitudinal loyalty and behavioral loyalty. The scale consists of a total of seven questions, such as, “In my opinion, working in this organization is the best choice.” Both dimensions of the scale demonstrated satisfactory internal consistency, with Cronbach’s alpha coefficients exceeding 0.70. Participants rated their responses on a 6-point Likert scale, with 1 indicating complete nonconformity and 6 indicating complete conformity. The Cronbach’s alpha coefficient for this scale in this study was 0.91.

*Control variables*: given that gender and age may influence communication patterns and authority acceptance, education level could correlate with technical comprehension and innovation adoption, while tenure directly affects experience accumulation and skill proficiency, this study controls for demographic differences between masters and apprentices, including gender, age, education level, and organizational tenure—to mitigate potential confounding effects from these individual factors (Matta et al., 2015).

## 4 Results

### 4.1 Validated factor analysis and discriminant validity

To assess the structural and discriminant validity of each variable, this study conducted a validated factor analysis of benevolent leadership, authoritative leadership, loyalty, and craftsman spirit. As shown in Table 1, the four-factor model demonstrated an optimal fit index, suggesting that the variables possess good discriminant validity.

**Table 1:** Results of structural validity and common method deviation tests for core variables.

Model	*χ* ^2^	df	χ^2^/df	RMSEA	CFI	TLI	SRMR
One-Factor Model: AL + BL + LO + CS	1515.76	90	16.84	0.22	0.49	0.41	0.18
Two-Factor Model: BL, AL + LO + CS	1088.26	89	12.23	0.18	0.64	0.58	0.15
Three-Factor Model: BL, AL, LO + CS,	689.12	87	7.92	0.14	0.79	0.74	0.11
Four-Factor Model: BL, AL, LO, CS,	270.07	84	3.22	0.08	0.93	0.92	0.05
Five-Factor Model: BL, AL, LO, CS, CMV	270.07	83	3.25	0.08	0.93	0.92	0.05

BL, Benevolent Leadership; AL, Authoritarian Leadership; LO, Loyalty; CS, Craftsman Spirit; CMV, Common Method Variable. Mark “+” indicates the coalition of variables.

### 4.2 Common method deviation test

First, the results of the Harman single-factor test showed that the variance explained by the first factor prior to rotation was 31.02%, falling below the threshold of 40%. Second, despite efforts to control for any unmeasured factors, the fit indicators of the five-factor model did not show any significant improvement (Xie and Yan, 2016). It can be concluded that the issue of common method bias among the variables examined in this study is not serious.

### 4.3 Descriptive statistics and correlation analysis

The mean, standard deviation, and correlation coefficient of the variables in this study are shown in Table 2. Notably, benevolent leadership exhibited significant and positive correlations with both loyalty (r = 0.34, *p* < 0.01) and craftsman spirit (r = 0.18, *p* < 0.01). Conversely, authoritarian leadership demonstrated significant negative correlations with loyalty (r = −0.15, *p* < 0.01) and craftsman spirit (r = −0.13, *p* < 0.05). Furthermore, there was a significant correlation between loyalty and craftsman spirit (r = 0.22, *p* < 0 0.01).

**Table 2:** Descriptive statistics and correlation analysis between variables.

Variables	M	SD	1	2	3
1. Benevolent leadership	4.18	1.17			
2. Authoritarian leadership	2.35	0.94	−0.25^**^		
3. Loyalty	4.14	1.08	0.34^**^	−0.15^**^	
4. Craftsman spirit	4.39	0.71	0.18^**^	−0.13^*^	0.22^**^

*Significantly correlated at the 0.05 level (two-tailed); **significantly correlated at the 0.01 level (two-tailed).

### 4.4 Hypothesis testing

The analysis of the proportion of consistent and inconsistent samples of masters’ benevolent-authoritarian leadership behaviors indicated that the inconsistent samples of accounted for 70.3%, surpassing the 10% criterion for evaluation. Consequently, in accordance with the guidelines of Edwards and Parry (1993), this study employed polynomial regressions and response surface analysis to construct regression equations and examine the hypotheses. Polynomial regression enables a comprehensive capture of the joint effects of two predictor variables by establishing a quadratic regression equation, while response surface analysis visually reveals the pattern of variation along both the “authoritarian-benevolent consistency line” and the “authoritarian-benevolent inconsistency line”.

The effect of benevolent-authoritarian leadership on craftsman spirit is detailed in Model 4 of Table 3. It was observed that the slope and curvature of the response surface along the interface along the consistency line (X = Y) were not statistically significant (slope = 1.38, *p* > 0.1; curvature = 1.21, *p* > 0.1). This suggests that the difference in the apprentice’s craftsman spirit between the high benevolence-high authority and the low benevolence-low authority leadership style was not significant, thereby failing to validate H1. This outcome may be attributed to the specific research sample drawn from highly regulated, high-intensity industries such as mechanical processing and geological exploration. Within such contexts, masters’ highly authoritarian behaviors may create a compounding effect with the inherent institutional pressures apprentices routinely face, resulting in perceived over control and constraint that triggers psychological reactance. Consequently, this suppresses creative expression and intrinsic motivation for excellence. In essence, within already highly standardized environments, excessive authoritarian intervention may exceed apprentices’ tolerance threshold, thereby negating the compensatory effect of benevolence.

**Table 3:** Polynomial regression results and response surface analysis.

Variables	Loyalty		Craftsman Spirit	
	Model 1	Model 2	Model 3	Model 4
Constant term	29.16^***^	26.92^***^	114.98^***^	110.68^***^
Control variables				
Gender differences	−0.04	0.15	−4.80^*^	−4.82^*^
Educational background differences	−1.07	−0.86	−0.17	0.05
Age differences	0.55	0.41	−0.84	−0.96
Tenure differences	−0.07	−0.02	−2.29^*^	−2.15^*^
Independent variables				
Benevolent leadership (X) b_1_		1.61^**^		2.59^*^
Authoritarian leadership (Y) b_2_		−0.12		−1.22
Benevolence-squared (X^2^) b_3_		0.15		0.50
Benevolence × Authority (X × Y) b_4_		−0.30		0.46
Authority-squared (Y^2^) b_5_		0.14		0.25
ΔR^2^	–	0.13^***^	–	0.04^***^
Response surface analysis				
Consistency line (X = Y)				
Slope (a_1_ = b_1_ + b_2_)		1.49		1.38
Curvature (a_2_ = b_3_ + b_4_ + b_5_)		−0.01		1.21
Inconsistency line (X = -Y)				
Slope (a_3_ = b_1_-b_2_)		1.73^*^		3.81^*^
Curvature (a_4_ = b_3_-b_4_ + b_5_)		−0.29		0.28

*N* = 343, ***p < 0.001, **p < 0.01, *p < 0.05.

Conversely, the slope coefficient of the response surface along the inconsistency line (X = −Y) was significantly positive (slope = 3.81, *p* < 0.05). This implies that, in comparison to the low benevolence-high authority leadership style, the apprentice’s craftsman spirit was higher in the high benevolence-low authority leadership style. Thus, H2 was supported. These findings strongly align with theoretical expectations derived from social exchange theory and provide robust empirical support from the context of Chinese mentorship systems. When masters exhibit high benevolence-low authority leadership, they essentially establish a high-quality social exchange relationship characterized by predominant benevolence and minimized cost. Apprentices perceive such guidance and care as sincere investments beyond contractual norms, consequently demonstrating reciprocal behavior through meticulous craftsmanship. Conversely, highly authoritarian behaviors, even when accompanied by limited benevolence, are often interpreted as instrumental control mechanisms, thereby confining the relationship to the “economic exchange” level and suppressing the activation of intrinsic motivation.

The effect of benevolent-authoritarian leadership on apprentice loyalty is presented in Model 2 of Table 3. It was observed that the slope and curvature of the response surface along the consistency line (X = Y) were not statistically significant (slope = 1.49, *p* > 0.1; curvature = −0.01, *p* > 0.1). This suggests that the difference of apprentice loyalty between the high benevolence-high authority and the low benevolence-low authority leadership styles was not significant. Conversely, the slope coefficient of the response surface along the inconsistency line (X = -Y) was significantly positive (slope = 1.73, *p* < 0.05). This implies that, in comparison to the low benevolence-high authority style, apprentice loyalty was higher in the high benevolence-low authority leadership style.

**Figure 2:**
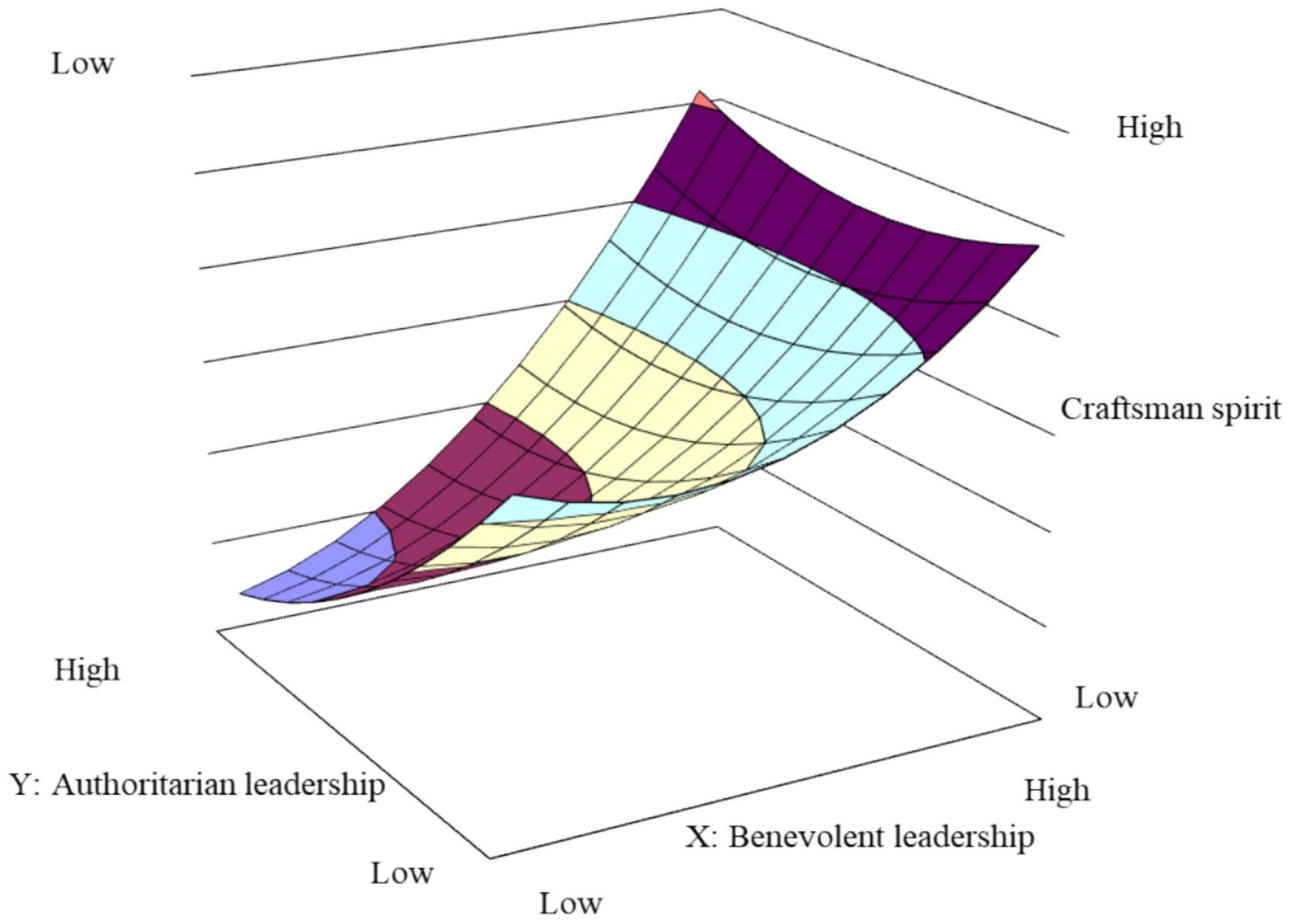
The effect of consistent benevolent-authoritarian leadership on craftsman spirit.

**Figure 3:**
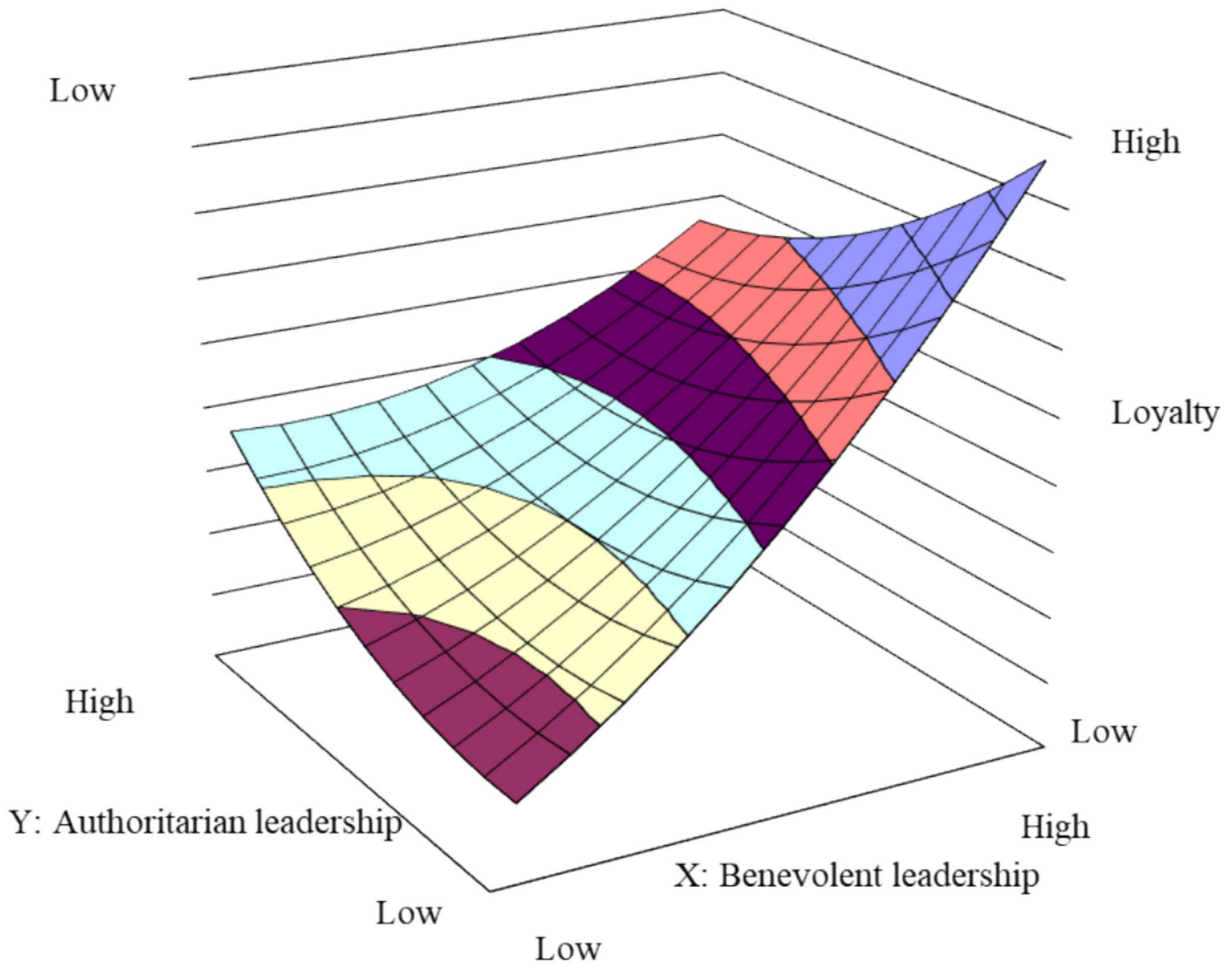
The effect of consistent benevolent-authoritarian leadership on apprentice loyalty.

To provide a visual representation of the (in)consistency of masters’ benevolent-authoritarian leadership behaviors on apprentices’ craftsman spirit and loyalty, this study has created three-dimensional models of the response surfaces. These are depicted in Figures 2, 3, using the data from Model 4 and Model 2 in Table 3, respectively. As shown in Figure 2, the inconsistency line extending from the “high authority-low benevolence” region (front-left) to the “high benevolence-low authority” region (back-right) demonstrates a statistically significant upward linear trend. This indicates that as the master’s benevolence progressively increases and surpasses their authoritarianism, the apprentice’s craftsman spirit shows corresponding enhancement. In contrast, the surface along the consistency line from low-low to high-high configurations remains relatively flat, suggesting that “high-high” leadership does not yield significantly greater craftsman spirit than “low-low” leadership. As shown in Figure 3, the relatively flat surface along the consistency line indicates no substantial difference in apprentice loyalty between “high-high” and “low-low” leadership conditions. However, the consistently ascending slope along the inconsistency line from “high authority-low benevolence” to “high benevolence-low authority” regions demonstrates that loyalty effectively increases when the master’s benevolent behaviors exceed their authoritarian behaviors.

Based on the findings presented above, this study employed a synthetic block variable to test the mediating effect of (in)consistency in masters’ benevolent-authoritarian leadership behaviors on apprentices’ craftsman spirit through apprentice loyalty. Based on the coefficients obtained from prior polynomial regression analysis, a composite block variable was constructed by synthesizing the five independent variables (Table 3) according to their respective weights. This composite block variable was subsequently incorporated as an integrated independent variable into the mediating effect model to verify the mediating mechanism. This analytical approach not only faithfully preserves the interactive nature of authoritarian-benevolent leadership as a dual-concept, but also effectively addresses collinearity issues among polynomial terms in structural equation modeling, thereby ensuring both statistical validity in testing mediating effects and theoretical completeness in interpretation. The results show that the *Benevolence-Authority* set-area variable significantly and positively influences apprentice loyalty (b = 1.00, *p* < 0.001), and apprentice loyalty, in turn, significantly influences craftsman spirit (b = 0.42, *p* < 0.05). H3 was supported as evidenced by the mediating effect of apprentice loyalty on the *Envision* set-area variable and craftsman spirit, yielding a coefficient of 0.42, with a 95% bias-corrected confidence interval of [0.23, 0.68]. This result was obtained after 5,000 repetitions of sampling.

## 5 Discussion and conclusion

Drawing upon the principles of SET, this study explored the mechanism underlying the impact of masters’ authoritarian-benevolent leadership behaviors on apprentices’ craftsman spirit. The results reveal that, when masters’ benevolent and authoritarian leadership behaviors were inconsistent, the improvement in apprentices’ craftsman spirit was more pronounced in the high benevolence-low authority leadership style compared to the low benevolence-high authority leadership style. Furthermore, it was established that apprentice loyalty played a mediating role between masters’ authoritarian-benevolent leadership behaviors and apprentices’ craftsman spirit. These findings contribute to a deeper understanding of the mechanisms through which masters’ authoritarian-benevolent leadership behaviors influence the apprentices’ craftsman spirit. They offer a theoretical foundation and practical insights on how to effectively foster and cultivate the craftsman spirit.

First, this study makes a significant theoretical contribution by revealing, through the context of Chinese mentorship systems, the effectiveness of the “high benevolence-low authority” leadership pattern, thereby establishing a meaningful dialogue with dual leadership theories developed in Western contexts. Western dual leadership theories emphasize the balanced integration of two complementary leadership behaviors, such as exploratory and exploitative leadership (Keller and Weibler, 2015) or transformational and transactional leadership (Schreuders and Legesse, 2012), as key drivers of work performance (Rosing et al., 2011). In contrast, our response surface analysis demonstrates that within the Chinese cultural context, benevolent and authoritarian leadership behaviors exhibit an asymmetric pattern, with the “high benevolence-low authority” configuration showing particularly strong effects in promoting apprentice loyalty and craftsman spirit. This finding challenges the equilibrium paradigm predominant in traditional dual leadership theories and highlights the cultural contingency of leadership effectiveness (House et al., 2004). Within the Chinese cultural logic that emphasizes “benevolence” “loyalty” and “reciprocity,” the relational bonds and social exchange relationships established through masters’ benevolent behaviors prove more effective in stimulating apprentices’ intrinsic motivation and emotional commitment than the normative constraints generated through authoritarian behaviors, thereby more successfully cultivating craftsman spirit. This research not only enriches understanding of how leadership behaviors influence craftsman spirit but also underscores the uniqueness and complexity of indigenous management practices, providing evidence from the Chinese context for developing more culturally inclusive leadership theories.

Furthermore, this study validates and extends the social exchange perspective by identifying apprentice loyalty as a mediating mechanism between masters’ authoritarian-benevolent leadership and craftsman spirit. The findings reveal that such leadership cultivates loyalty through social exchange processes, which subsequently serves as a fundamental psychological driver for developing craftsman spirit. This not only unveils the internal black box between dual leadership and workplace outcomes (Chan et al., 2013), but more significantly, establishes loyalty as the central psychological mechanism through which external leadership influences are transformed into employees’ internal pursuits within Chinese mentorship systems. Crucially, the high benevolence-low authority configuration demonstrates optimal effectiveness, indicating significant differences in how masters’ benevolent versus authoritarian behaviors elicit reciprocal responses from apprentices. Specifically, intensive benevolent investments trigger stronger loyalty reciprocation that partially substitutes or compensates for deficiencies in authoritative behaviors. This finding reveals the unequal potency of different resources in reciprocal exchanges, demonstrating that socioemotional resources (benevolence) exert stronger driving forces than normative resources (authority) in constructing high-quality exchange relationships within Chinese mentorship contexts.

This study offers practical implications for cultivating craftsman spirit in organizations. First, organizations should recognize the value of authoritarian-benevolent leadership in workplace dynamics. While competent instructors are readily available, true masters who cultivate character are rare. Organizations should implement personality assessments and behavioral observations during master selection to identify individuals capable of exercising balanced authoritarian-benevolent leadership. Such leadership enables apprentices to perceive genuine care from their masters while understanding the constructive intent behind authoritative guidance. Second, organizations should provide authority soft-skilling training for masters. Through scenario-based workshops analyzing organizational cases, masters can learn to focus authoritative behaviors on technical standards and quality requirements while granting apprentices appropriate autonomy in work methods and innovation attempts. This approach fosters a leadership pattern combining “technical rigor” with “managerial flexibility,” ultimately enhancing apprentice performance and stimulating innovative behaviors. Furthermore, organizations should acknowledge loyalty’s crucial role in promoting craftsman spirit. To effectively enhance apprentice loyalty, firms can establish institutionalized master-apprentice matching systems and regular communication mechanisms to establish a good relationship. These measures provide stable interactive foundations for developing social exchange relationships between mastering relationships and apprentices. Simultaneously, implementing emotional bank account strategies that encourage masters to consistently accumulate relational capital through professional guidance, personal care, and career development support can strengthen these bonds.

## 6 Limitations and further research

The limitations of this study are as follows. First, while the empirical measurement procedure employed data collection methods from multiple sources and time points, which helped mitigate homologous bias, it is essential to acknowledge that the self-assessment methods used to measure apprentices’ loyalty in this study may be influenced by various subjective factors. Future research could consider a combination of self-assessment and other-assessment approaches to compensate for any shortcomings stemming from apprentices’ self-assessment. Second, While this study focuses on authoritarian-benevolent leadership within Chinese mentorship systems, future research should urgently pursue cross-cultural comparative investigations. By collecting master-apprentice dyads from diverse cultural contexts such as North America and Europe, the applicability and effect variations of authoritarian-benevolent leadership across different cultural settings should be examined. Such comparative work would help clarify the cultural boundaries of the high benevolence-low authority pattern’s effectiveness originally identified in the Chinese context. Variations in the effects of different leadership behaviors on apprentices’ craftsman spirit may exist. Future research should explore the influence of other ambidextrous leadership styles, such as open-closed ambidextrous leadership behaviors (Wang et al., 2022), to provide more comprehensive organizational recommendations for cultivating apprentices’ craftsman spirit. Lastly, this study concentrated on the mediating mechanisms through which masters’ authoritarian-benevolent leadership behaviors affect apprentices’ craftsman spirit via loyalty. Previous studies have noted that employees’ characteristics influence the practical outcomes of masters’ leadership behaviors. Future research should explore whether there are boundary conditions that affect these positive factors and how to further enhance their positive effects.

## Data Availability

The data that support the findings of this study are available from the corresponding authors upon request. Requests to access these datasets should be directed to Huanhuan Zhang, d202281620@hust.edu.cn.
